# Interprofessional education of final-year medical students and trainee anaesthesia assistants (IPAPA) – a project report on an interprofessional training sequence on induction of anaesthesia

**DOI:** 10.3205/zma001856

**Published:** 2026-06-15

**Authors:** Gregor Massoth, Maria Wittmann, Andrea Tölle, Andreas Jurkscheit, Mark Coburn, Götz Fabry, Johannes Biedermann, Achilles Delis

**Affiliations:** 1 University Hospital Bonn, Department of Anaesthesiology and Intensive Care Medicine, Bonn, Germany; 2 University Hospital Bonn, School of Anaesthesia Assistance, Bonn, Germany; 3 University of Freiburg, Institute of Medical Psychology and Medical Sociology, Freiburg, Germany

**Keywords:** interprofessional communication, TeamSTEPPS® 3.0, simulation, team training, medical education, final year, trainee anaesthesia assistants

## Abstract

**Introduction:**

Interprofessional collaboration is considered a key determinant of patient care quality and safety, yet it remains only partially embedded in the curricula of healthcare professions. The IPAPA project, developed at the University Hospital Bonn, addresses this gap through the design of a simulation-based interprofessional training programme for final-year medical students in their elective anaesthesiology rotation and trainee anaesthesia assistants, based on the evidence-informed TeamSTEPPS^®^ programme.

**Objective:**

This project report aims to describe the curricular development, implementation, and instructional design of the IPAPA teaching concept and to contextualise initial experiences from the accompanying evaluation.

**Methods:**

The teaching intervention was developed in accordance with Kern’s six-step approach to curriculum development. Interprofessional learning objectives were derived from the National Competence-Based Learning Objectives Catalogue for Medicine and the German training and examination regulations for anaesthesia assistants and operating theatre technicians (ATA-OTA-APrV), and translated into an adapted TeamSTEPPS^®^ 3.0-based teaching and learning concept with simulation-based components. Implementation was complemented by an accompanying evaluation.

**Results:**

The IPAPA concept, comprising eight teaching units, was integrated as a mandatory course into both the final-year elective rotation in anaesthesiology and the training programme for trainee anaesthesia assistants. Initial qualitative feedback from participants suggests high acceptance of the format, particularly with regard to the practical simulations and the structured interprofessional communication. Joint participation of both professional groups was described as realistic and conducive to mutual role understanding.

**Conclusion:**

This project report illustrates how an interprofessional, simulation-based team training programme can be developed and implemented within an anaesthesiology training context. IPAPA thus contributes to the structured integration of interprofessional teaching formats and may serve as a model for comparable educational settings.

## Zusammenfassung

**Einleitung:**

Interprofessionelle Zusammenarbeit gilt als wesentlicher Faktor für Patient:innenversorgung, ist jedoch in der Ausbildung medizinischer Berufsgruppen bislang nur begrenzt curricular verankert. Das am Universitätsklinikum Bonn entwickelte IPAPA-Projekt adressiert diesen Bedarf durch die Konzeption eines simulationsbasierten interprofessionellen Trainings für PJ-Studierende im Wahltertial Anästhesiologie und Anästhesietechnische Assistenz-Auszubildende auf Basis des evidenzbasierten TeamSTEPPS^®^-Programms.

**Zielsetzung:**

Ziel des Projektberichts ist die Darstellung der curricularen Entwicklung, Implementierung und didaktischen Ausgestaltung des IPAPA-Lehrkonzepts sowie die Einordnung erster begleitender Evaluationserfahrungen.

**Methodik:**

Die Entwicklung der Lehrveranstaltung erfolgte entlang der sechs Schritte der Curriculumsentwicklung nach Kern. Interprofessionelle Lernziele wurden aus dem Nationalen Kompetenzbasierten Lernzielkatalog Medizin und der Ausbildungs- und Prüfungsordnung ATA-OTA abgeleitet und in ein angepasstes TeamSTEPPS^®^ 3.0- basiertes Lehr-/Lernkonzept mit simulationsgestützten Anteilen überführt. Die Implementierung wurde durch eine begleitende Evaluation ergänzt.

**Ergebnisse:**

Das IPAPA-Konzept wurde mit acht Unterrichtseinheiten als verpflichtende Lehrveranstaltung in das Praktisches Jahr-Wahltertial Anästhesiologie sowie die Ausbildung zur Anästhesietechnischen Assistenz-Ausbildung integriert. Erste qualitative Rückmeldungen der Teilnehmenden deuten auf eine hohe Akzeptanz des Formats hin, insbesondere hinsichtlich der praxisnahen Simulationen und der strukturierten interprofessionellen Kommunikation. Die gemeinsame Teilnahme beider Berufsgruppen wurde als realitätsnah und förderlich für das gegenseitige Rollenverständnis beschrieben.

**Schlussfolgerung:**

Der Projektbericht zeigt exemplarisch, wie ein interprofessionelles, simulationsbasiertes Teamtraining im anästhesiologischen Ausbildungskontext curricular entwickelt und implementiert werden kann. IPAPA leistet damit einen Beitrag zur strukturierten Integration interprofessioneller Lehrformate und kann als Modell für vergleichbare Ausbildungskontexte dienen.

## 1. Introduction

Effective collaboration between different professional groups is regarded as essential for high-quality and safe patient care, as medical errors in many cases are attributable to human failure and deficient team processes [1], [2]. To prevent potential apathy towards teamwork and to sustainably establish patient-centred interprofessional care, early implementation of interprofessional educational formats has been recommended [3]. As recent studies have shown, interprofessional teaching in medical education in Germany is still only marginally embedded in curricula; many faculties continue to rely primarily on existing monoprofessional teaching formats that have only partially and selectively been transformed into interprofessional offerings [4].

The project “InterProfessional education of final-year medical students and trainee anaesthesia assistants” (IPAPA) at the University Hospital Bonn directly addresses this structural gap in educational practice through interprofessional training. The project is based on the Team Strategies and Tools to Enhance Performance and Patient Safety (TeamSTEPPS^®^) 2.0 (subsequently 3.0) programme developed by the Agency for Healthcare Research and Quality of the U.S. Department of Health and Human Services [5], and adapts its communication and teamwork tools for an anaesthesiology-specific interprofessional training format involving final-year medical students and trainee anaesthesia assistants at the University Hospital Bonn. TeamSTEPPS^®^-based approaches are already used locally in other interprofessional training contexts, for example in patient safety training in perinatal care [6].

The aim of this evidence-based programme is to optimise team performance, and it has been successfully implemented in a variety of clinical and academic contexts [7]. Studies have shown that the integration of TeamSTEPPS^®^ into interprofessional simulation training can result in significant improvements in team communication and collaboration, for example among medical students and nursing trainees [8], [9].

Numerous studies have demonstrated that simulation and interprofessional team training in the perioperative setting are associated with relevant positive clinical outcomes. These include, among others, reduced postoperative mortality, lower complication rates related to central venous catheters, and higher success rates for regional anaesthesia [10]. In addition, systematic reviews have shown that intraoperative teamwork interventions sustainably improve team communication, non-technical skills, and the early identification of latent safety risks in particular [11], [12]. Although most studies do not focus on anaesthesia-specific complication rates as an isolated endpoint, the consistently reported effects on team processes and patient-relevant outcomes underline the importance of interprofessional, simulation-based educational formats for patient safety in the anaesthesiology context.

The aims of the IPAPA project are:

1. Promotion of interprofessional teamwork and communication: Final-year medical students and trainee anaesthesia assistants are specifically trained to act jointly as a team, to reflect on their profession-specific roles, and to apply communication skills safely and effectively in the clinical context, particularly in critical situations.
2. Strengthening reflective capacity and professional development: Participants are to be enabled to engage in continuous self-reflection and personal development with regard to their interprofessional competencies. At the same time, the project aims to support the professionalisation of medical and nursing educators in simulation-based interprofessional teaching.
3. Establishing an interprofessional teaching and learning culture to improve patient safety: By creating a community of practice with trained multipliers (“change agents”) and by preparing, in the long term, for an interprofessional training ward (IPSTA) in anaesthesiology, a sustainable structure is to be created to strengthen communication and teamwork culture in the operating theatre and thereby improve clinical outcomes for patients.

## 2. Project description

The development of the project was guided by Kern’s six-step approach to curriculum development [13]. Within a participatory and dialogical development process, representatives of the Department of Anaesthesiology and Intensive Care Medicine, the School for Anaesthesia Assistance at the University Hospital Bonn, and the Dean of Studies Office of the Faculty of Medicine at the University of Bonn were involved. The aim was to identify current challenges as well as potential areas for innovation in the education and training of the professional groups involved [14]. This process revealed a need for the implementation of interprofessional teaching and for the targeted qualification of teaching staff in the fields of anaesthesiology education and trainee anaesthesia assistant training at the local site. To support the structural and methodological-didactic development of the course concept, close collaboration was established with the “Bonn Network for Interprofessional Teaching” at the Faculty of Medicine of the University of Bonn.

### 2.1. Step 1: Problem identification and general needs assessment

A substantial body of empirical evidence shows that interprofessional teaching formats can positively influence participants’ knowledge, skills, and attitudes [15]. This assessment is supported by various national regulations and initiatives. Both the Masterplan for Medical Education 2020 and the training and examination regulations for anaesthesia assistants and operating theatre technicians (ATA-OTA-APrV), as well as statements issued by the German Society for Medical Education, emphasise the need for the systematic integration of interprofessional educational content [16], [17], [https://www.gesetze-im-internet.de/ata-ota-aprv/BJNR229510020.html]. The ATA-OTA-APrV already allocates more than 120 teaching hours to this content in theoretical and practical instruction. It may also be assumed that these topics will become examination-relevant for medical students within the framework of the reform of the German medical licensing regulations ([https://nklm.de/zend/menu], chapter: VIII.3. Interprofessionelle Kompetenzen Lernziele).

For more than a decade, however, this need for interprofessional education has been highlighted not only in regulatory frameworks but repeatedly in scientific analyses and position papers, while actual implementation continues to lag clearly behind stated expectations [4], [18], [19], [20].

Against this background, the needs assessment conducted within the IPAPA project included a systematic identification of relevant learning objectives for both participating professional groups. In doing so, both the requirements of NKLM 2.0 and the ATA-OTA-APrV were taken into account (see table 1 (Tab. 1)) [https://www.gesetze-im-internet.de/ata-ota-aprv/BJNR229510020.html, [https://nklm.de/zend/menu].

### 2.2. Step 2: Targeted needs assessment

At the University Hospital Bonn, medical education is likewise still delivered predominantly in a monoprofessional manner. The Faculty of Medicine has therefore launched the funding programme “Learning with and from one another” to establish a longitudinal curriculum on “Interprofessional Competencies and Patient Safety”; the project described here contributes one component to this broader initiative. In addition, the Graduate Profile in Medicine (2020) explicitly formulates interprofessional aspects in several Entrustable Professional Activities (EPAs), defining close collaboration between different health professions as a prerequisite for competence-based medical practice [16]. This creates an additional curricular mandate to systematically anchor interprofessional teaching opportunities.

**Figure 1:**
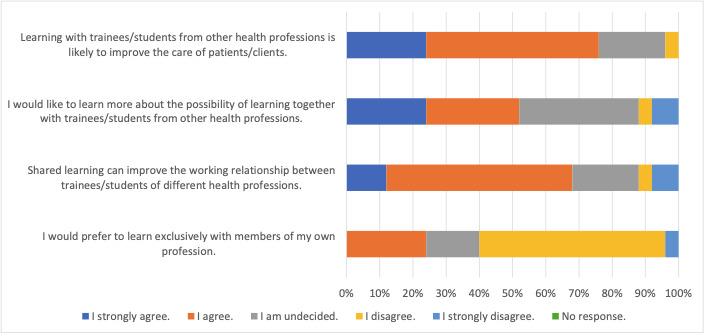
Interest of trainee anaesthesia assistants (N=11) and medical students in the anaesthesiology rotation (N=14) in interprofessional teaching

Although separate curricula exist in anaesthesiology for final-year medical students in the elective rotation and for trainee anaesthesia assistants, there have so far been no joint teaching activities. A preliminary survey of trainee anaesthesia assistants (N=11) and medical students in the anaesthesiology rotation (N=14) showed that both groups had had little prior experience with interprofessional teaching. At the same time, there was strong interest in more interprofessional learning opportunities and low approval of purely monoprofessional teaching (see figure 1 (Fig. 1)).

These findings underline the need for structural adjustments at the Bonn site in order to promote interprofessional collaboration in a targeted way and thereby sustainably improve quality of care.

### 2.3. Step 3: Learning objectives

The overarching aim of the IPAPA teaching project is to prepare participants specifically for their future role as active members of interprofessional treatment teams. Central to this is the reflection on profession-specific roles during induction of anaesthesia and the practice-oriented training of cooperative behaviour in an interprofessional context. These competencies are taught both in the simulation setting and in routine clinical practice during standard assignments involving induction of anaesthesia. Against this background, the project team developed specific learning objectives for the training units (see table 2 (Tab. 2)). [https://www.ahrq.gov/teamstepps-program/index.html]. In addition, participants’ prior knowledge and respective level of experience were systematically taken into account [21].

### 2.4. Step 4: Teaching and learning concepts

The teaching and learning concept of the IPAPA project was developed on the basis of the defined interprofessional learning objectives (step 3) and follows an action-oriented and experiential didactic logic [22]. The aim was to integrate the teaching of practical competencies related to induction of anaesthesia with interprofessional communication and teamwork aspects, thereby reflecting the complexity of real clinical work situations.

Conceptually, the curriculum is oriented towards the evidence-based TeamSTEPPS^®^ programme, which specifically addresses non-technical competencies such as communication, leadership, situational awareness, and mutual support, and whose effectiveness in interprofessional educational settings has been demonstrated [5], [7], [8], [9], [22], [23]. As TeamSTEPPS^®^ 3.0 was designed for a broad range of healthcare professions, the content, priorities, and didactic implementation were specifically adapted to the needs of final-year medical students and trainee anaesthesia assistants in the context of induction of anaesthesia. To use the limited course time efficiently, elements of the original curriculum that were less directly relevant to practice (e.g. change management) were deliberately omitted.

The systematic alignment of interprofessional learning objectives, teaching and learning content, and didactic methods in the sense of Kern’s curriculum development model is shown in table 3 (Tab. 3) and is explained below by describing the specific design of the individual teaching modules.

#### 2.4.1. Didactic introduction and interprofessional familiarisation (introductory day)

The introductory day forms the didactic starting point of the teaching concept and serves to establish a shared professional, communicative, and interprofessional learning foundation. In addition to a moderated introductory round and a structured pre-evaluation, expectations towards the respective other professional group, individual training status, and prior experiences with interprofessional learning are explored. This phase is intended to make implicit role perceptions and responsibilities visible and to initiate initial negotiation processes within the team. The deliberate confrontation with differing perspectives and experiential worlds promotes perspective taking and the development of a shared understanding of roles as a basis for interprofessional collaboration [24], [25].

Subsequently, central practical and professional principles of induction of anaesthesia are taught in an interprofessional manner. These include the structured preparation of induction of anaesthesia, application of basic monitoring, insertion of a peripheral venous catheter, use of safety-relevant checklists (e.g. WHO checklist, PANAMA), induction-related drugs, and fundamentals of airway management, including the management of unexpectedly difficult airways. These contents are intentionally not taught separately by profession but are developed jointly in order to explicitly reflect differences in responsibilities, roles, and experience levels (see table 3 (Tab. 3)).

A further focus of the introductory day is preparation for simulation-based learning. Participants are familiarised with the aims, principles, and limitations of simulation, including the importance of a protected learning environment, clear rules for feedback, and the separation of learning and assessment situations. Observation priorities for the simulations (e.g. workplace preparation, communication, situational awareness) are defined in advance to enable structured reflection during the subsequent debriefings [26], [27].

#### 2.4.2. Simulation as an integrative learning format

The simulation-based components form the core of the teaching concept. Realistic full-scale simulation scenarios were developed to represent typical situations during induction of anaesthesia, including elective standard inductions as well as unexpected respiratory or haemodynamic incidents. The scenarios were designed to address both practical-professional and interprofessional-communicative learning objectives simultaneously (cf. table 3 (Tab. 3)).

Final-year medical students and trainee anaesthesia assistants work together in clinically realistic roles, analogous to actual collaboration in the operating theatre. Role allocation is intentionally kept flexible to allow situational leadership and dynamic task distribution. The simulations are supervised by experienced anaesthesiology instructors, who remain in an observational role during the scenarios.

The didactic focus is less on the technical perfection of individual measures than on interprofessional coordination, the application of structured communication strategies (e.g. SBAR, check-back), and joint decision-making under time pressure. In this context, SBAR supports the structured handover of complex clinical information, whereas check-back ensures mutual confirmation of critical information and thus contributes to the development of a shared mental model [28], [29].

The combination of interprofessional teaching and simulation-based learning formats is considered particularly suitable for the practice-oriented training of team processes, communication, and collaboration [8], [9], [26], [27], [30].

#### 2.4.3. Feedback, reflection and transfer phases

Each simulation is followed by a structured instructor-led debriefing based on established principles of simulation-based teaching. The aim is the systematic reflection on both professional aspects and teamwork and communication processes. Participants analyse their own actions as well as team interaction and discuss alternative courses of action. Through the targeted reflection of differing perspectives, a reflective learning process is initiated that extends beyond purely technical skills [24], [30].

Between modules, specific links to clinical practice are established: participants are encouraged to consciously apply key communication strategies and team principles in routine clinical work and to revisit these experiences in subsequent reflection phases. Formal prescribed learning tasks for the practice phases were deliberately omitted to allow flexible integration of transfer into the clinical workflow.

#### 2.4.4. Curricular alignment and methodological orientation

The selection of teaching and learning methods was made in close alignment with the defined learning objectives (cf. table 3 (Tab. 3)). Affective learning objectives (e.g. role understanding, mutual support) are primarily addressed through interactive seminar components, simulation, and reflection; cognitive learning objectives (e.g. understanding of structured communication) through theory-based introduction and application in simulation scenarios; and psychomotor learning objectives through practical training in induction of anaesthesia within the simulation setting. The systematic interweaving of these elements ensures that interprofessional competencies are not taught additively, but integratively.

#### 2.4.5. Structural sequence of the teaching concept

**Figure 2:**
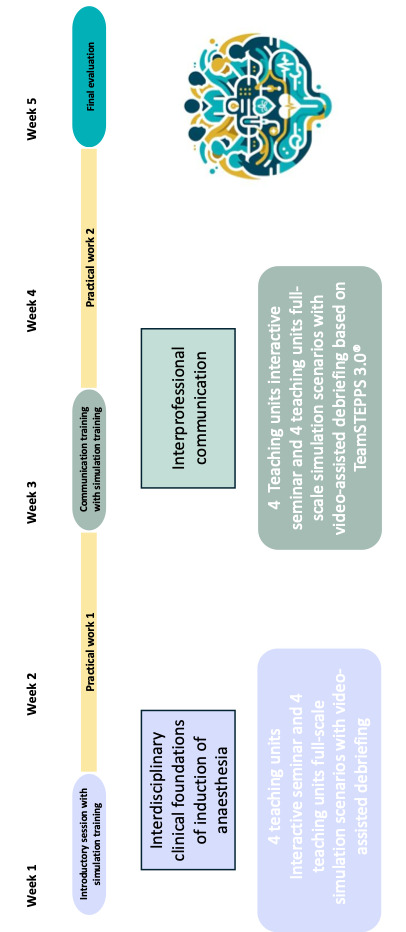
Overall concept of the IPAPA teaching project for the intervention group (authors’ own illustration); the control group received the communication training with simulation after the final evaluation

The training concept is divided into two consecutive modules with a total duration of five weeks (see figure 2 (Fig. 2)). The first module comprises an introductory session with interactive seminar teaching and an initial simulation training, while the second module consists of an advanced communication training based on the TeamSTEPPS^®^ 3.0 model with expanded simulation training and defined incident scenarios. This structure reflects the typical duration of interprofessional educational interventions and allows a gradual deepening of the learning content [24], [26], [27].

### 2.5. Step 5: Implementation

The implementation of the IPAPA teaching project followed the prerequisites for successful curricular implementation as described by Kern. The project was approved by the Ethics Committee of the Faculty of Medicine Bonn (reference no. 061/23 EP); the staff council of the University Hospital Bonn likewise raised no objections to the planned implementation.

During the implementation phase, a systematic analysis of available resources was conducted. This included personnel resources (faculty and anaesthesia assistants educators), temporal resources (integration of course units into final-year rotations and the trainee anaesthesia assistants training phases), infrastructure (skills lab rooms and simulation technology), and financial support through the university teaching programme. The design and organisational preparation of the teaching project extended over a period of approximately eight months; the overall concept is shown in figure 2 (Fig. 2).

Responsibilities within the project team were clearly defined. Overall academic and organisational coordination was led by the project leader, while the operational delivery of the course units was jointly undertaken by faculty and anaesthesia assistants educators. All involved teaching staff were specifically prepared for their role in advance, including an introduction to the TeamSTEPPS^®^ concept and to didactic principles of simulation-based interprofessional teaching.

To ensure an adequate and as homogeneous as possible level of competence among participants for the simulation-based training, final-year medical students in the elective anaesthesiology rotation and trainee anaesthesia assistants at an advanced stage of training (second and third year) were selected for participation. Organisationally, the intervention was conducted before the start of each new final-year rotation. Mandatory course dates were established for both professional groups for the introductory and communication training sessions; participating faculty and educators were released from their regular clinical duties for the duration of the training sessions. The infrastructural prerequisites were ensured by early reservation of suitable skills lab rooms and provision of the required simulation technology.

The first successful implementation of the IPAPA course took place in two iterations between May and July 2023, each with trainee anaesthesia assistants and final-year medical students. Group sizes were determined by the size of the graduating anaesthesia assistants class of 2023. Since then, the teaching format has been offered several times per year on a regular basis, allowing each final-year medical student cohort and each anaesthesia assistants class the opportunity to participate. These structural and organisational measures form the basis for stable and reproducible implementation of the IPAPA concept and are closely linked to the challenges described in the discussion section, particularly regarding resource demands, coordination of interprofessional learning groups, and infrastructural requirements.

### 2.6. Step 6: Evaluation

The evaluation of the course concept was conducted as part of an accompanying scientific study using a mixed-methods approach (Kirkpatrick levels 1-3) [31], [32]. Written informed consent was obtained from all participants. The present project report focuses on the curricular development and implementation of the IPAPA teaching concept. The qualitative evaluation is therefore intentionally presented only in summary form below and serves to illustrate perceived learning effects. A detailed qualitative analysis with full category development and in-depth interpretation will be reported in a separate publication.

In addition to participant evaluation, the implementation process of the teaching concept was documented throughout the project. This included recorded team and steering group meetings of the interprofessional project team, structured reflections following the simulation sessions, and organisational project documents (e.g. group composition, time schedules, room and resource availability). The challenges described in the discussion section are based on the integrative analysis of these implementation data and on recurring themes from the qualitative interviews.

To assess training effects, intervention and control groups were formed. However, for the qualitative evaluation presented in this project report, the interview data were not analysed separately by group, as the aim of this initial qualitative analysis was not to demonstrate comparative intervention effects but rather to capture the general acceptance, perception, and feasibility of the interprofessional teaching concept from the participants’ perspective. Accordingly, interview statements from both groups were analysed jointly. Comparative qualitative analyses between groups will be addressed in a separate in-depth publication.

For organisational reasons, participants were assigned in chronological sequence, as implementation of the intervention involved increased personnel and logistical demands; accordingly, the control groups were conducted first, followed by the intervention groups. The interval between the two modules in the intervention groups was approximately 2 weeks. Final evaluation was conducted in both groups approximately 4 weeks after the start of the intervention. During the observation period, the control group received no TeamSTEPPS^®^-specific training and only participated in the introductory session with simulation training after completion of the final evaluation. This ensured that all participants were able to benefit equally from the TeamSTEPPS^®^ training after the completion of the study.

The summary qualitative evaluation presented here focuses on the results of 22 semistructured interviews (9 in the control group, including 3 trainee anaesthesia assistants and 6 final-year medical students; 13 in the intervention group, including 5 trainee anaesthesia assistants and 8 final-year medical students), based on the following research questions: *“Can interprofessional training influence learning and collaboration between different professional groups in anaesthesiology?”* “Is this form of learning accepted?” “Does training in interprofessional communication exert an additional influence?”

The interviews were transcribed and analysed using Mayring’s qualitative content analysis [33]. Categories were developed deductively on the basis of the research questions and inductively from the material. Coding was carried out systematically, and the results were subsequently summarised interpretatively.

The qualitative analysis of the semistructured interviews was conducted across groups and included participants from both the control and intervention groups. The aim of this presentation is not an in-depth qualitative analysis or a comparison between groups, but rather an illustrative presentation of central perceptions regarding acceptance, feasibility, and perceived learning effects of the interprofessional teaching format. A full qualitative analysis with systematic category development and deeper interpretation will be reported in a separate publication.

Across all interviews, recurring thematic areas emerged. Participants particularly described:

- the importance of clear and structured interprofessional communication,
- simulation-based teaching components as practical and memorable,
- a deeper understanding of the respective roles and competencies,
- a generally appropriate perceived level of challenge despite heterogeneous prior experiences,
- and indications of possible transfer into clinical routine, while also acknowledging structural constraints.

To illustrate these themes, one participant stated: “Communication is the be-all and end-all.”

Another participant emphasised the practical value of the simulations: “The simulation is what stays with you the most.”

These exemplary statements are intended solely to illustrate the thematic breadth and do not replace a detailed qualitative presentation of results.

In addition, several quantitative instruments were used, the results of which are reported in detail elsewhere [34], [35], [31], [32], [36].

## 3. Discussion

To the best of our knowledge, no teaching projects have yet been published in which the TeamSTEPPS^®^ 3.0 training concept has been specifically applied to final-year medical students and trainee anaesthesia assistants. The IPAPA project therefore provides an example demonstrating that interprofessional teaching formats in the anaesthesiology context can be designed in a way that is both didactically feasible and educationally effective.

The IPAPA project was deliberately guided by Kern’s six-step model of curriculum development, as it offers a clearly structured framework that is well established in medical education for the systematic development, implementation, and evaluation of curricular interventions [13]. At the same time, the project showed that the model requires adaptation, particularly in an interprofessional and simulation-based context, as also described by Kern [13]. Individual steps did not proceed in a strictly linear manner, but rather iteratively and in close feedback loops with the participating stakeholders. In particular, the fine-tuning of learning objectives, the design of simulation scenarios, and organisational decisions were revised multiple times in order to accommodate the differing training logics of final-year medical students and trainee anaesthesia assistants. These experiences underline that Kern’s model should be understood less as a rigid sequence and more as a heuristic framework for curricular development processes.

A central didactic decision of the IPAPA project was the deliberate integration of practical-professional content related to induction of anaesthesia with interprofessional communication and teamwork aspects within the same simulation scenarios. In contrast to educational formats that teach technical and non-technical skills separately, an integrated approach was chosen here in order to reflect the real complexity of anaesthesiological work situations. Simulation proved to be a suitable learning environment in which to make this integration tangible, but at the same time imposed increased demands on didactic design, debriefing, and participants’ competence levels. In particular, the balance between professional overload and educationally productive challenge emerged as a continuous didactic task. Positive participant feedback suggests that this integrative approach was experienced as practical and meaningful, but also highlights that such an approach requires increased resources and preparation.

At the same time, the development, planning, and implementation process revealed several key challenges on the basis of accompanying implementation documentation, structured team reflections, and recurring participant feedback, all of which should be considered when adapting comparable formats. These included in particular:

- *Curricular and organisational barriers,* for example the coordination of final-year medical students and trainee anaesthesia assistants with differing timetables, rotation structures, and learning objectives. This was compounded by differing group sizes, as trainee anaesthesia assistants participated in fixed training classes, whereas the number of final-year medical students varied and was generally smaller. This required flexible composition of training groups and the conscious decision not to establish fixed dyads between final-year medical students and trainee anaesthesia assistants. Instead, interprofessional collaboration in changing constellations was fostered with the aim of developing team competencies independently of fixed pairings, in line with routine clinical practice, while ensuring that both professions were represented simultaneously in all training sessions.
- *Resource demands, especially the need for project coordination,* additional personnel for in situ evaluations, and the personnel and temporal challenge of locating participants for data collection within the clinical environment (e.g. in the operating theatre).
- *Infrastructure issues,* such as the availability of suitable skills lab rooms and technical equipment for simulation-based units.
- *The importance of preparatory measures, *particularly a structured introductory session to ensure that all participants possess sufficient professional foundations and prior clinical experience to participate effectively and safely in interprofessional simulation training.
- *The close curricular integration of profession-specific and interprofessional learning objectives, *especially with regard to communication, teamwork, and joint clinical decision-making.

These aspects make clear that successful implementation of interprofessional teaching formats in a clinically practice-oriented setting requires not only didactic expertise, but also structural and personnel resources as well as close coordination between the professional groups involved.

The qualitative findings reported here should be understood as an exploratory complement to the curricular description. The aim was not to provide a complete qualitative presentation of results, but rather to contextualise initial evaluation experiences within the implementation of the teaching concept. Positive participant feedback on the realistic composition of the training groups and on the practical teaching of key communication competencies underscores the high relevance of the format. Through simulation-based training in combination with seminar teaching, the IPAPA project contributes to the targeted promotion of core interprofessional competencies in the setting of induction of anaesthesia and thereby supports the development of a respectful and cooperative team culture – an important factor in addressing the growing shortage of skilled personnel in anaesthesiology.

The accompanying qualitative evaluation should be interpreted critically in light of its aims and scope. The interviews primarily sought feedback on acceptance of the learning sequence, perceived learning processes, and initial experiences with transfer into routine clinical practice and can therefore largely be assigned to Kirkpatrick levels 1 (reaction) and 2 (learning) [37]. It was not possible within the scope of this project report to systematically assess sustained behavioural transfer or patient-relevant outcomes (levels 3 and 4). In addition, it should be noted that the qualitative analysis presented in this publication is intentionally summary in nature and exploratory in character. The results therefore do not serve as proof of effectiveness, but rather as a contextualised interpretation of implementation and learning experiences and as a basis for further, more in-depth analyses in a separate publication.

At present, it remains unclear to what extent the competencies taught will lead to sustained behavioural changes in the clinical setting in view of increasing demands in anaesthesiology practice, including flexible teams and increasingly complex patients. This question can only be answered conclusively through the detailed analysis of the extensive quantitative and qualitative accompanying evaluation, which is currently being prepared for a separate publication.

From a future perspective, it appears reasonable to examine the transferability of individual modules or didactic elements to other specialties and to evaluate their integration into existing curricula of interprofessional educational concepts.

## 4. Conclusion

Interprofessional team training programmes such as IPAPA can make a substantial contribution to the promotion of teamwork competencies, communicative confidence, and mutual role understanding in clinical education. The structured integration of simulation-based scenarios and the targeted focus on interfaces of collaboration represent key success factors in this regard.

## Notes

### Author contributions

Gregor Massoth developed the conceptual framework of the teaching project together with Achilles Delis and Götz Fabry as a project and master’s thesis within the postgraduate Master of Medical Education (MME-D) programme at Heidelberg University. All authors provided feedback during development of the concept. Johannes Biedermann, Maria Wittmann, Andrea Tölle, Andreas Jurkscheit, and Gregor Massoth delivered the course sessions. Gregor Massoth was supported by Götz Fabry in the interpretation of the data. All authors contributed to the conception of the manuscript. Gregor Massoth, Johannes Biedermann, Achilles Delis, and Maria Wittmann drafted the manuscript and received critical feedback from all authors. Johannes Biedermann and Dr Achilles Delis contributed equally to this work and share joint senior authorship.

### Authors’ ORCIDs

- Gregor Massoth: [0000-0002-4973-2208]
- Maria Wittmann: [0000-0003-4786-7712]
- Mark Coburn: [0000-0002-7930-0270]
- Goetz Fabry: [0000-0002-5393-606X]
- Achilles Delis: [0000-0003-0363-8643]

### Funding

The teaching project was funded by the programme “Learning with and from one another” of the Faculty of Medicine at the University of Bonn, Bonn, Germany.

### Use of AI-assisted tools

The AI-assisted application ChatGPT (OpenAI, version GPT-4.0, as of April 2025) was used to support the linguistic optimisation and structuring of individual text passages as well as the preparation of preliminary summaries and outline suggestions. Full responsibility for the content remains with the author.

## Competing interests

The authors declare that they have no competing interests. 

## Figures and Tables

**Table 1:**
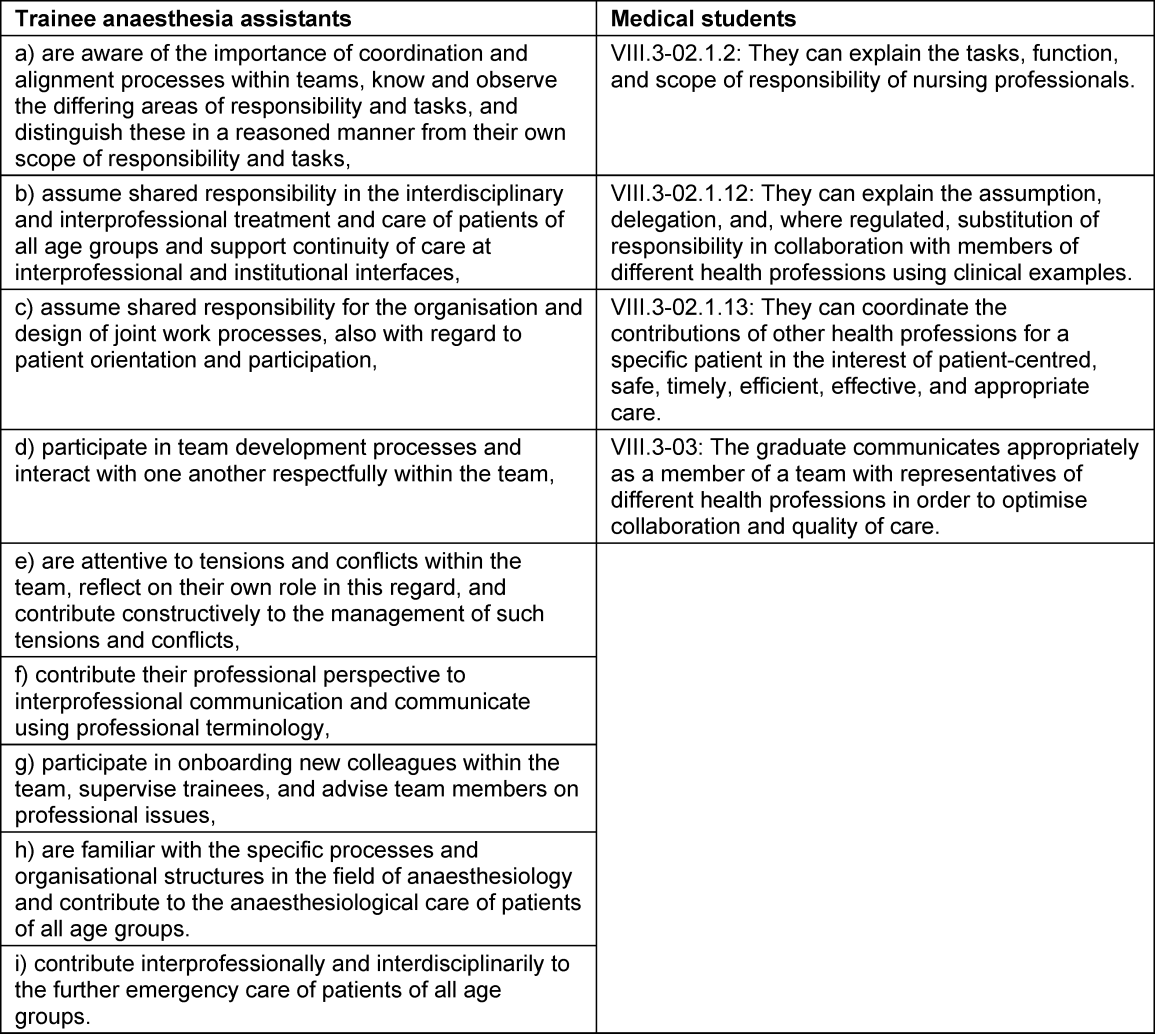
Interprofessional learning objectives derived from the ATA-OTA-APrV and NKLM 2.0

**Table 2:**
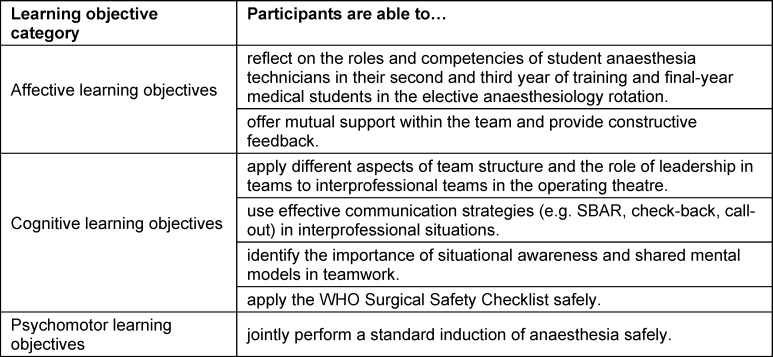
Specific learning objectives of the IPAPA teaching project

**Table 3:**
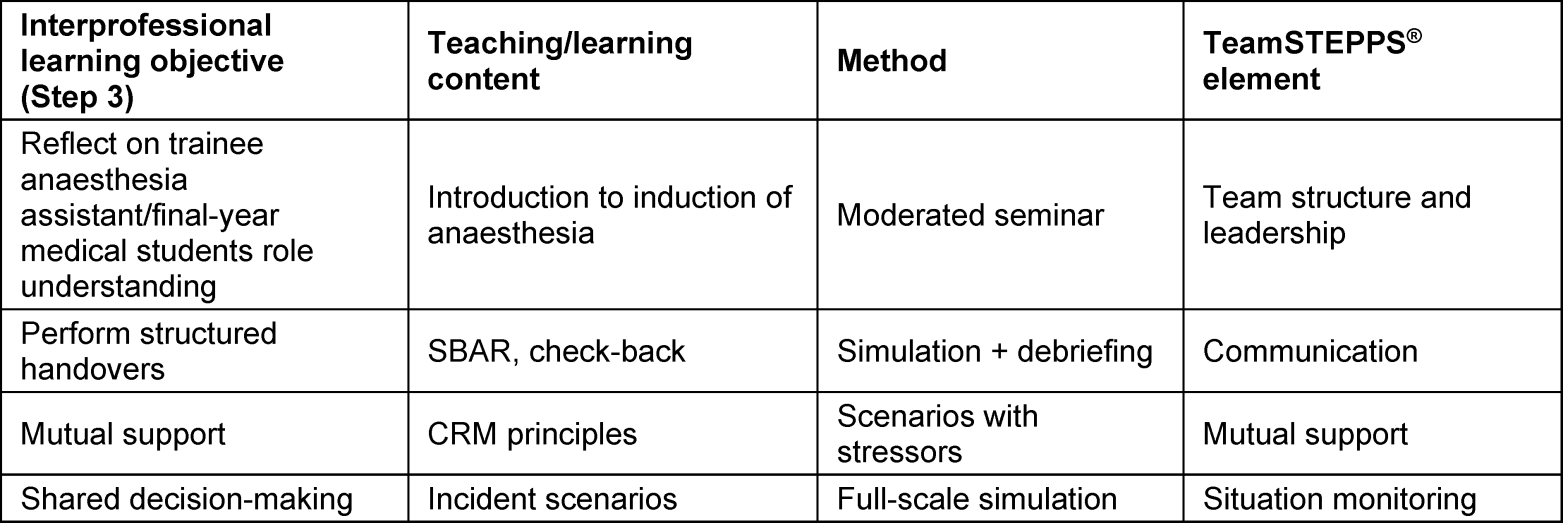
Alignment of interprofessional learning objectives with teaching/learning content and methods
